# Current Evidence of the Efficacy and Safety of Neoadjuvant EGFR-TKIs for Patients With Non-small Cell Lung Cancer

**DOI:** 10.3389/fonc.2021.608608

**Published:** 2021-05-24

**Authors:** Xiaoshun Shi, Xiaoying Dong, Jianxue Zhai, Xiguang Liu, Di Lu, Zhen Ni, Hua Wu, Kaican Cai

**Affiliations:** Department of Thoracic Surgery, Nanfang Hospital, Southern Medical University, Guangzhou, China

**Keywords:** neoadjuvant therapy, EGFR-TKI, meta-analysis, NSCLC, surgery

## Abstract

**Purpose:**

Epidermal growth factor receptor tyrosine kinase inhibitors (EGFR-TKIs) have been indicated to be an effective treatment for advanced EGFR-mutant NSCLC. However, the neoadjuvant application of EGFR-TKIs in resectable NSCLC needs further investigation. Here, we aimed to evaluate the efficacy and safety of neoadjuvant EGFR-TKIs for lung cancer.

**Methods:**

Published studies on neoadjuvant EGFR-TKIs in NSCLC were identified in PubMed, Web of Science, and EMBASE until June 1, 2020. Data on surgical rates, objective response rates (ORRs), pathologic responses, and adverse event (AE) rates were retrieved for proportional meta-analysis.

**Results:**

In total, 7 enrolled studies involving 129 EGFR-TKI-sensitive NSCLC patients were included in this analysis. The overall surgical rate in these studies was 95% (95% CI: 83% to 100%), with an ORR of 48% (95% CI: 39% to 57%) in the population with EGFR-TKI-sensitive mutations, whereas the ORR including wild-type EGFR patients was 28% (95% CI: 14% to 44%). The rate of grade 1-2 AEs was 69% (95% CI: 41% to 91%) but with an acceptable rate of grade 3-4 AEs of 0% (95% CI: 0% to 5%). The pooled rates of rash and diarrhea were 56% (95% CI: 31% to 79%) and 25% (95% CI: 6% to 51%), respectively. The impact of neoadjuvant EGFR-TKIs on survival remains inconclusive.

**Conclusions:**

Neoadjuvant EGFR-TKIs showed objective responses in approximately half of EGFR-sensitive NSCLC patients with a tolerable adverse effect profile. The favorable impact of neoadjuvant EGFR-TKIs on NSCLC needs more evidence for validation, such as the comparison of survival improvement between EGFR-TKIs and chemotherapy. The efficacy of neoadjuvant next-generation EGFR-TKIs in clinical trials remains unclear.

## Introduction

Surgery is an effective treatment for non-small cell lung cancer (NSCLC), but the 5-year overall survival (OS) rates of patients with stage II and IIIA disease are only 65% and 41%, respectively (1). Even when the tumors in these patients have been radically resected, micrometastasis may exist before surgery and is considered to be the main factor causing postoperative local or distant recurrence. In addition to the elimination of micrometastases, preoperative systemic treatment could result in tumor shrinkage and decreased lymph node enlargement, therefore reducing the TNM stage and tumor burden and facilitating the surgical procedure. Therefore, the optimal neoadjuvant therapy should be to reduce tumor burden without delaying the scheduled operation and have fewer adverse effects. Studies have shown that the use of neoadjuvant chemotherapy can improve the OS of NSCLC patients (2). Although targeted therapy led by epidermal growth factor receptor tyrosine kinase inhibitors (EGFR-TKIs) and immunotherapy led by PD-1 inhibitors have been proven to be effective treatments in advanced NSCLC, the application of those reagents as neoadjuvant therapy for lung cancer other than chemotherapy is still at the exploration stage.

For the large group of patients with EGFR gene mutations, the administration of EGFR-TKIs is preferred (3, 4). Compared to the controversial molecular markers for the prediction of the efficacy of immunotherapy, the limited application of EGFR-TKIs in patients with EGFR wild-type NSCLC and the low abundance of EGFR mutations (5) are widely accepted. However, the design of previous neoadjuvant EGFR-TKI clinical trials did not distinguish between populations that were sensitive and those with wild-type mutations (6, 7). In more recent studies, the safety and efficacy of neoadjuvant EGFR-TKI therapy have been more focused on populations with EGFR-TKI-sensitive mutations (8, 9). In theory, sensitive mutations may improve the efficacy of neoadjuvant EGFR-TKIs, but there is currently little evidence to support this hypothesis.

In addition to the above clinical trial designs, which are based on changes in EGFR mutation status, a more detailed design taking clinical staging into consideration is needed. From 2009 to 2016, clinical trials tried to cover a broad spectrum of TNM stages, including patients from stage I to stage IV, and wild-type EGFR status (6, 10). Since 2016, the study designs have tended to focus on NSCLC patients with stage II and stage III disease (8, 9) with EGFR-sensitive mutations. In addition, a comparison of neoadjuvant EGFR-TKIs and chemotherapy (11, 12) suggests that neoadjuvant EGFR-TKIs can improve patient prognosis compared with chemotherapy. Nevertheless, the chemotherapy group in the study by Zhong et al. (11) was administered gemcitabine plus cisplatin, while in the study by Xiong et al. (12), cisplatin-based doublet chemotherapies including vinorelbine, gemcitabine, paclitaxel, docetaxel or pemetrexed were administered. In the cases of limited sample sizes and different chemotherapy combinations contributing as a confounding factor, the level of evidence for this conclusion needs to be improved by adding more results in future studies.

Though a series of phase II trials on neoadjuvant EGFR-TKIs for NSCLC have been reported, the safety and efficacy of neoadjuvant EGFR-TKIs, especially in subgroups of EGFR mutation status or TNM staging, remain unclear. Considering that these trials have great potential to change current neoadjuvant practice in lung cancer surgery, we performed a meta-analysis incorporating the results of the surgical rates, clinical responses, pathologic responses, toxicities, and prognoses to evaluate the safety and efficacy of neoadjuvant EGFR-TKI therapy.

## Materials and Methods

We prospectively registered the protocol for this study in the International Prospective Register of Systematic Reviews (PROSPERO number: CRD42020187031**).** We reported the analysis by following the Meta-Analysis of Observational Studies in Epidemiology (MOOSE) standards (**Table S1**) and the Preferred Reporting Items for Systematic Reviews and Meta-Analyses (PRISMA) statement.

### Literature Retrieval

We performed a literature search in PubMed, Web of Science, and EMBASE until June 1, 2020. We used the following combination of keywords: “NSCLC”, “EGFR-TKI”, “neoadjuvant”, “preoperative”, and drug names of EGFR-TKIs. The detailed literature search criteria are listed in the supplementary files. We also performed an additional search through Google Scholar. Two authors (XY Dong and JX Zhai) removed the duplicated literature independently. Only studies reported in English were included.

### Inclusion and Exclusion Criteria

We included studies based on the following criteria: (I) studies reported NSCLC patients with neoadjuvant EGFR-TKI therapy, and any generation of EGFR-TKIs was permissive; and (II) the surgical rate, objective response rate (ORR), and rate of adverse events (AEs) were available. Studies with the following characteristics were excluded from this meta‐analysis: (I) studies from the same institutions or research group, studies with a close timeframe, and the same clinical trials (only the largest patient population was included); (II) comments, letters, and reviews; (III) incomplete data that are unable to be used for statistical analysis, such as studies that do not provide the ORR and rate of AEs; and (IV) case reports or studies with sample sizes less than 10.

### Quality Assessment and Data Extraction

We (JX Zhai, XG Liu, Z Ni) used the Newcastle-Ottawa Scale to assess all the included studies (**Table S2**). Funnel plots were used to assess publication bias for outcomes reported by a minimum of 3 studies.

Data on the surgical rate, ORR, rate of AEs, and survival outcome were extracted by JX Zhai and Z Ni independently. In this study, the assessment of the surgical rate was limited to patients with EGFR-sensitive mutations. Other measurements included patients with wild-type EGFR in the expanded analysis. We reached a final consensus if discrepancies occurred.

### Statistical Analysis

The surgical rates of patients with EGFR-sensitive mutations receiving neoadjuvant EGFR-TKIs were calculated by the actual number of surgeries divided by the total number of patients. ORR was defined as the sum of the complete response plus partial response divided by the total number of included patients. Similarly, the number of pathological responses and grade 1-2 and grade 3-4 AEs were retrieved from the included literature and then transformed into rates by dividing by the total number of included patients. Survival data were retrieved by the methods reported by Tierney et al. (13). We performed a normality test for each rate in the proportional meta-analysis based on the raw rate, log transformation, logit transformation, arcsine transformation, and Freeman-Tukey double arcsine transformation to determine which method was best for the pooled analysis (**Table S3**). Finally, we applied the Freeman-Tukey double arcsine transformed proportion in the pooled analysis. As reported in our previous studies (14), heterogeneity was measured by the Cochran Q test and I^2^ value. We reported values from the random effects model for studies with potential heterogeneity; otherwise, values from the fixed effects model were reported. All analyses were performed using R version 3.5.1, in which the proportional meta-analysis was performed with the “meta” package and the meta-regression analysis was performed with the “metafor” package. We considered a statistical test with a P value < 0.05 as significant.

## Results

### Features of the Eligible Studies

**Figure 1:**
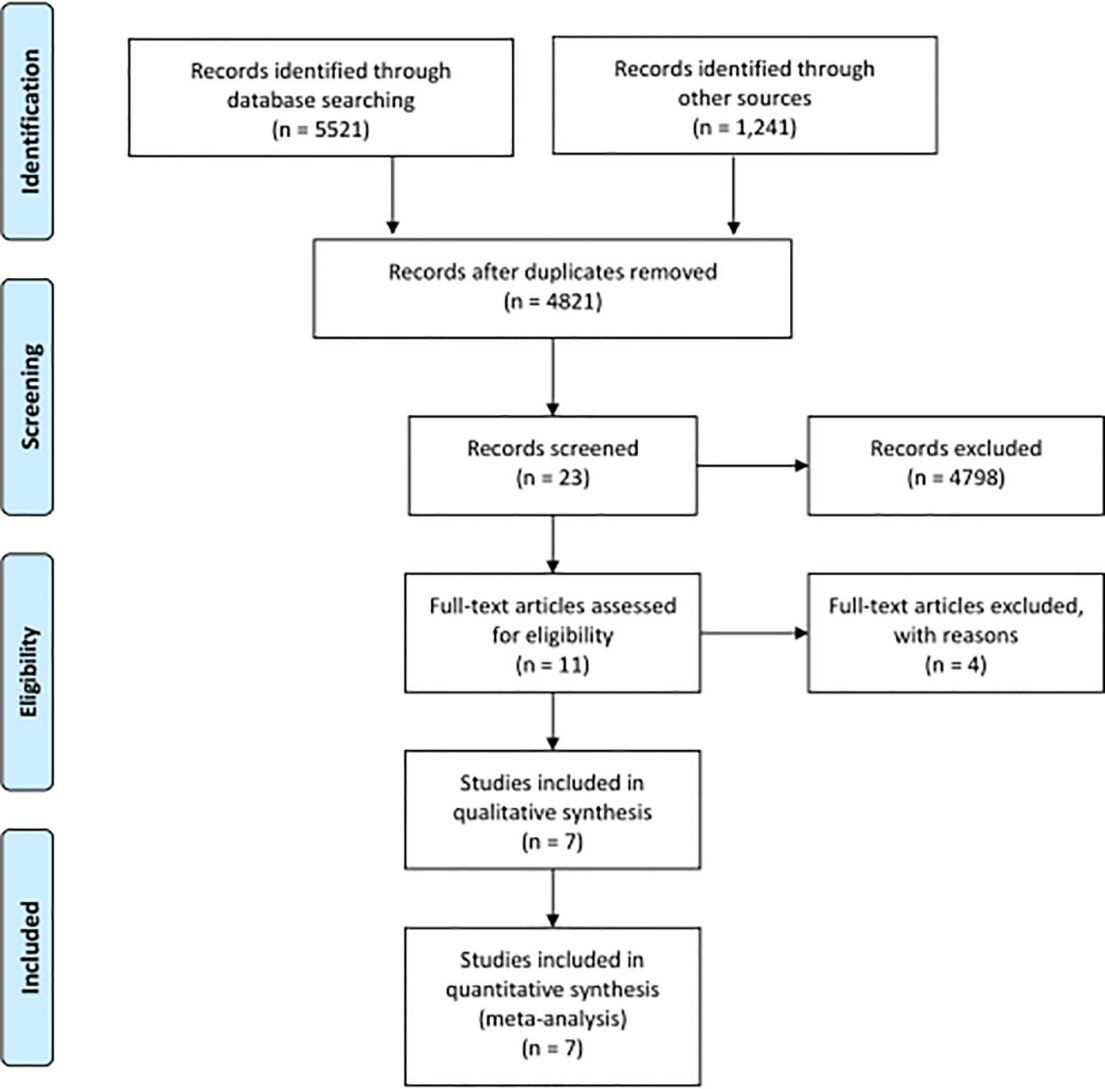
The PRISMA 2009 flow diagram.

We identified records based on the search strategy and finally enrolled 7 studies involving 129 NSCLC patients with clear EGFR-sensitive mutation status out of a total of 312 patients, with a summary provided in **Table 1**. The PRISMA 2009 flow diagram is shown in **Figure 1**. In this analysis, the exact number of patients with EGFR-TKI-sensitive mutations was not available in two studies and was partially available in two studies, so we included only the selected number in the relevant analysis.

**Table 1:** Characteristics of the included studies.

Author	Public Year	Location	Research Center	Study Year	Trialphase	Stage	Group	Sample size	Age	EGFR-TKI	Preoperative treatment
Yang Zhang	2020	China	Single	2013.8-2015.10	II	II-IIIA	Single arm	35	57 (52-63) median (IQR)	Gefitinib	250 mg of oral gefitinib daily for 42 days
Liwen Xiong	2018	China	Single	2011.7- 2014.6	II	IIIA-N2	Single arm	19	59 (33–74) median (range)	Erlotinib	150 mg per day for 56 days
Wenzhao Zhong							2019					China	multiple	2011.12-2017.12	II	IIIA-N2	Erlotinib	37	59 (32-73) median (range)	Erlotinib	Patients received erlotinib 150 mg/d, 42 days
	Gemcitabine + Cisplatin	35	58 (33-76) median (range)	Chemo	gemcitabine 1,250 mg/m^2^ plus cisplatin 75 mg/m^2^											
Tao Wang	2016	China	Single	2011.12-2014.12	Retrospective	IA–IIIA	Single arm	67	59 (37–78)median	Icotinib	125 mg thrice daily until the day before surgery
Eva E. Schaake	2012	Netherlands	multicenter	2006.12- 2010.11	II	I-IV	Single arm	60	64 (37 -74) median (range)	Erlotinib	150 mg once daily for 3 weeks^*^
Lara-Guerra	2009	Canada	Single	NA	II	I	Single arm	36	65 (38 -81) median (range)	Gefitinib	250 mg orally once daily for up to 28 days
Haura	2010	USA	Single	NA	Pilot Study	IA-IIIB	Single arm	23	70 median	Gefitinib	250 mg daily for 4 weeks before surgical resection.

Chemo, chemotherapy; *: dose was reduced due to toxicity.

### Neoadjuvant EGFR-TKIs Are Feasible

**Figure 2:**
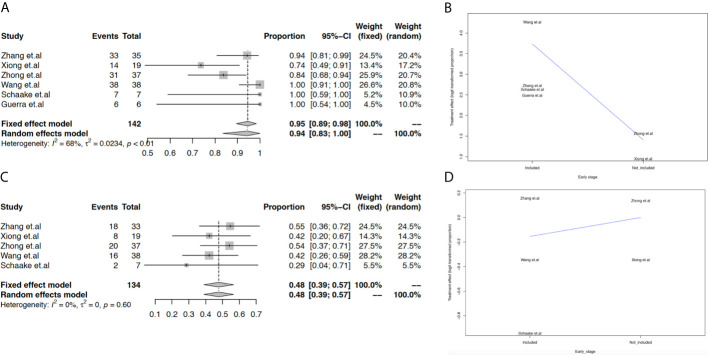
Meta-analysis and meta-regression analysis of surgical rate and ORR. The pooled surgical rate in the population with EGFR-TKI-sensitive mutations **(A)**; meta-regression analysis of the surgical rate based on different advanced stages **(B)**; the pooled ORR in the population with EGFR-TKI-sensitive mutations **(C)**; meta-regression analysis of ORR based on different TNM stages **(D)**.

We evaluated the feasibility of neoadjuvant EGFR-TKIs based on the pooled estimation of the surgical rate, pathologic response, ORR, rate of stable disease, and rate of grade 3-4 AEs. Overall, the surgical rate in the population with EGFR-TKI-sensitive mutations was 94% (95% CI: 83% to 100%, **Figure 2A**). Additionally, meta-regression analysis indicated that the surgical rate could decrease in the advanced stage population (**Figure 2B**). Other important measurements for the justification of neoadjuvant therapy are tumor response. The cutoff of 50% tumor necrosis and no more than 10% viable tumor cells were both considered as pathological response in this study. Only three studies reported pathological response, with a pooled estimated rate of 20% (95% CI: 6% to 38%, **Figure S1**). In our analysis, the ORR in the population with EGFR-TKI-sensitive mutations was 48% (95% CI: 39% to 57%, **Figure 2C**), while the ORR in the overall population including patients with wild-type EGFR status decreased to 28% (95% CI: 14% to 44%, **Figure S2**). In populations with EGFR-TKI-sensitive mutations, studies including early-stage NSCLC may decrease the ORR of neoadjuvant EGFR-TKIs (**Figure 2D**). Of note, the rate of stable disease in the population with EGFR-TKI-sensitive mutations was 45% (95% CI: 36% to 53%, **Figure S3**), which could more likely occur in early-stage NSCLC (**Figure S4**).

**Figure 3:**
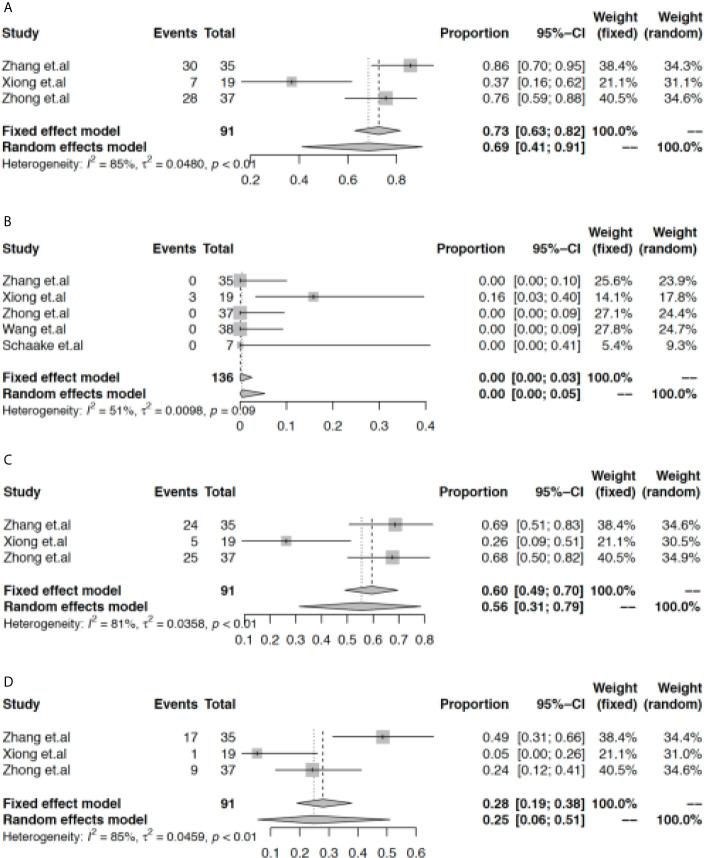
Meta-analysis of the rate of adverse effects. The pooled rate of grade 1-2 AEs **(A)**, grade 3-4 AEs **(B)**, rash **(C)**, and diarrhea **(D)**.

Next, we found that the rate of grade 1-2 AEs reached 69% (95% CI: 41% to 91%, **Figure 3A**), and the rate of grade 3-4 AEs was 0% (95% CI: 0% to 5%, **Figure 3B**). In the population with EGFR-TKI-sensitive mutations, rash and diarrhea were the most common adverse effects, with pooled rates of 56% (95% CI: 31% to 79%, **Figure 3C**) and 25% (95% CI: 6% to 51%, **Figure 3D**), respectively. However, the current evidence did not support the increased rate of rash (45%, 95% CI: 29% to 62%, **Figures S5** and **S6**) or diarrhea (22%, 95% CI: 12% to 34%, **Figures S7** and **S8**) when patients with wild-type EGFR were included. In the subgroup analysis, the rate of rash was higher while the rate of diarrhea was lower in the early TNM stage subgroup when neoadjuvant EGFR-TKIs were used in the overall population (**Figures S9** and **S10**).

### The Impact of Neoadjuvant EGFR-TKIs on Survival

Detailed survival data were not available in the majority of current publications. Only two studies reported survival data related to neoadjuvant EGFR-TKIs compared with neoadjuvant chemotherapy. Zhang et al. (8) reported a median disease-free survival (DFS) of 33.5 months (95% CI, 19.7-47.3), while Xiong et al. (9) reported a median DFS of 10.5 months (95% CI, 7.7-29.9). For the comparison of the survival outcomes of neoadjuvant EGFR-TKIs versus neoadjuvant chemotherapy, Zhong et al. (11) reported that the median progression-free survival (PFS) and OS were significantly longer with erlotinib than with gemcitabine plus cisplatin chemotherapy (HR, 0.39; 95% CI, 0.23 to 0.67; P < 0.001; and HR, 0.77; 95% CI, 0.41 to 1.45; P = 0.417). Similar to these results, in one excluded study, Xiong et al. (12) reported that erlotinib may have a survival benefit compared with cisplatin-based doublet chemotherapy in terms of DFS (HR, 0.51; 95% CI, 0.13 to 2.01; P =0.39) and OS (HR, 0.45; 95% CI, 0.04 to 5.54; P =0.12), but a significant difference was not found. However, the chemotherapy arm in this study included vinorelbine, gemcitabine, paclitaxel, docetaxel or pemetrexed with limited participants (n=16). Moreover, different adjuvant therapy regimens and surgical procedures (segmentectomy, lobectomy, and pneumonectomy) may impose different impacts on individual survival. Therefore, the contribution of neoadjuvant EGFR-TKIs to survival remains inconclusive.

### Assessment of Publication Bias

All publication bias was analyzed by Egger’s test and visualized by funnel plots, as shown in **Figure S11**. No significant publication bias was found.

## Discussion

Currently, neoadjuvant therapy based on chemotherapy has been proven to be effective (2, 15). The unsatisfactory overall response, adverse effects, and sometimes delay of surgery or inoperability, especially in the middle and late stages of NSCLC, require a more effective adjuvant treatment option. In this analysis, neoadjuvant EGFR-TKI therapy was shown to be a potential alternative for NSCLC patients with EGFR-TKI-sensitive mutations.

Compared with the overall response rates ranging from 50 to 70% depending on the combination (16) in neoadjuvant chemotherapy studies, the 48% ORR in the population with EGFR-TKI-sensitive mutations in this analysis seems to be acceptable. When considering the 45% stable disease rate in the population with EGFR-TKI-sensitive mutations including those with early-stage NSCLC, we hypothesize that the small EGFR-sensitive mutation tumors have relatively low abundances of EGFR mutations. Therefore, the improvement is not apparent. Of note, patients with advanced TNM stages may benefit more from neoadjuvant EGFR-TKIs, while early-stage patients may not benefit much, suggesting that there would be an optimal cutoff TNM stage to achieve a better neoadjuvant EGFR-TKI outcome. Furthermore, this study also suggests that the ORR can be significantly reduced with mixed wild-type mutation studies. Although we were unable to reanalyze the EGFR mutation status of subgroups of patients in some of the previous neoadjuvant chemotherapy studies, Zhong et al. (11) reported that the ORR for neoadjuvant erlotinib is better than that of gemcitabine plus cisplatin chemotherapy (54.1% versus 34.3%) in the EGFR-sensitive population. This finding suggests that this choice of chemotherapy in the EGFR-sensitive population as a neoadjuvant therapy could be inferior to the use of EGFR-TKIs. Of course, more definite decision making depends on more evidence from clinical trials in the future.

Another indicator of whether a drug is suitable for neoadjuvant therapy is the occurrence and level of the AEs. Although preoperative chemotherapy has advantages, its toxicity and side effects cannot be ignored. In addition to affecting liver and kidney functions, chemotherapy drugs also present toxicities in the cardiovascular and nervous systems. Although the occurrence of side effects is closely related to the dose and combination of chemotherapy drugs, in general, EGFR-TKIs have fewer side effects. Similar to the findings of previous EGFR-TKI studies (17), the most common side effects in neoadjuvant EGFR-TKI studies were rash and diarrhea. Although more than half of the patients had grade 1-2 AEs, fortunately, only a small number of patients had grade 3-4 AEs, possibly avoiding the accumulating toxicity from the long-term use of EGFR-TKIs in previous clinical trials. This confers one of the essential factors to ensure that the surgery is performed as scheduled.

There are many limitations in current neoadjuvant EGFR-TKI studies. First, this study has not been included results from ongoing clinical trials on the neoadjuvant therapy with next-generation EGFR-TKIs, such as afatinib (NCT04201756) or osimertinib (NCT03433469). Second, most of the current clinical trials based on patients with EGFR-TKI-sensitive mutations are from China, and more evidence from the Caucasian population is needed. The current studies inconsistently reported the effect of neoadjuvant EGFR-TKIs on survival (only 2 out of 7 studies), so it is not rational to perform a pooled analysis for this outcome. Further studies on this limitation are warranted. It is unknown whether the combination of neoadjuvant EGFR-TKIs and chemotherapy or immunotherapy achieves a better response rate and prolongs NSCLC survival. Finally, for the intrinsic few studies have been reported in and the methods of controlling were different among studies, the heterogeneity cannot be ignored. An updated meta-analysis is needed in the future.

We first provided pooled estimates of the surgical rate, response rate, and drug toxicity rate in patients receiving neoadjuvant EGFR-TKIs. Our analysis revealed that EGFR-TKIs are a promising neoadjuvant option for NSCLC patients with EGFR-TKI-sensitive mutations. Potential factors that affect these estimates were also investigated. Our findings indicate that neoadjuvant EGFR-TKIs could be more effective in NSCLC patients with EGFR-TKI-sensitive mutations than in those with wild-type EGFR.

## Data Availability Statement

The original contributions presented in the study are included in the article/**Supplementary Material**. Further inquiries can be directed to the corresponding author.

## Author Contributions

Conceptualization: KC and XS. Data collection and literature screening: XD, JZ, XL, and ZN. Data curation: XS, XD, and HW. Data analysis: XS. Funding acquisition: XS and KC. Methodology: XS and XD. Writing—original draft: DL, JZ and XS. Writing—review and editing: DL, ZN, XD, and XL. All authors contributed to the article and approved the submitted version.

## Funding

The work is partially supported by the Research Initiative Fund of Southern Hospital 2018 (C1051325) and the National Natural Science Foundation of China (81902319), the Major Science and Technology Planning Project of Guangdong Province (2017B020226005), and the Science and Technology Program of Guangzhou City (201903010003).

## Conflict of Interest

The authors declare that the research was conducted in the absence of any commercial or financial relationships that could be construed as a potential conflict of interest.

## References

[1] Goldstraw P Chansky K Crowley J Rami-Porta R Asamura H Eberhardt WE. The IASLC Lung Cancer Staging Project: Proposals for Revision of the TNM Stage Groupings in the Forthcoming (Eighth) Edition of the TNM Classification for Lung Cancer. J Thorac Oncol (2016) 11:39–51. 10.1016/j.jtho.2015.09.009 26762738

[2] NSCLC Meta-analysis Collaborative Group. Preoperative Chemotherapy for non-Small-Cell Lung Cancer: A Systematic Review and Meta-Analysis of Individual Participant Data. Lancet (2014) 383:1561–71. 10.1016/s0140-6736(13)62159-5 PMC4022989 24576776

[3] Maemondo M Inoue A Kobayashi K Sugawara S Oizumi S Isobe H. Gefitinib or Chemotherapy for non-Small-Cell Lung Cancer With Mutated EGFR. N Engl J Med (2010) 362:2380–8. 10.1056/NEJMoa0909530 20573926

[4] Fukuoka M Wu YL Thongprasert S Sunpaweravong P Leong SS Sriuranpong V. Biomarker Analyses and Final Overall Survival Results From a Phase III, Randomized, Open-Label, First-Line Study of Gefitinib Versus Carboplatin/Paclitaxel in Clinically Selected Patients With Advanced non-Small-Cell Lung Cancer in Asia (Ipass). J Clin Oncol (2011) 29:2866–74. 10.1200/jco.2010.33.4235 21670455

[5] Zhou Q Zhang XC Chen ZH Yin XL Yang JJ Xu CR. Relative Abundance of EGFR Mutations Predicts Benefit From Gefitinib Treatment for Advanced non-Small-Cell Lung Cancer. J Clin Oncol (2011) 29:3316–21. 10.1200/jco.2010.33.3757 21788562

[6] Lara-Guerra H Waddell TK Salvarrey MA Joshua AM Chung CT Paul N. Phase II Study of Preoperative Gefitinib in Clinical Stage I non-Small-Cell Lung Cancer. J Clin Oncol (2009) 27:6229–36. 10.1200/jco.2009.22.3370 19884551

[7] Schaake EE Kappers I Codrington HE Valdés Olmos RA Teertstra HJ van Pel R. Tumor Response and Toxicity of Neoadjuvant Erlotinib in Patients With Early-Stage non-Small-Cell Lung Cancer. J Clin Oncol (2012) 30:2731–8. 10.1200/jco.2011.39.4882 22753915

[8] Zhang Y Fu F Hu H Wang S Li Y Hu H. Gefitinib as Neoadjuvant Therapy for Resectable Stage II-IIIA non-Small Cell Lung Cancer: A Phase II Study. J Thorac Cardiovasc Surg (2020) 16(2):434–42.e2. 10.1016/j.jtcvs.2020.02.131 32340810

[9] Xiong L Li R Sun J Lou Y Zhang W Bai H. Erlotinib as Neoadjuvant Therapy in Stage IIIA (N2) EGFR Mutation-Positive non-Small Cell Lung Cancer: A Prospective, Single-Arm, Phase II Study. Oncologist (2019) 24:157–e64. 10.1634/theoncologist.2018-0120 30158288 PMC6369937

[10] Haura EB Sommers E Song L Chiappori A Becker A. A Pilot Study of Preoperative Gefitinib for Early-Stage Lung Cancer to Assess Intratumor Drug Concentration and Pathways Mediating Primary Resistance. J Thorac Oncol (2010) 5:1806–14. 10.1097/JTO.0b013e3181f38f70 PMC4120062 20881637

[11] Zhong WZ Chen KN Chen C Gu CD Wang J Yang XN. Erlotinib Versus Gemcitabine Plus Cisplatin as Neoadjuvant Treatment of Stage IIIA-N2 EGFR-Mutant non-Small-Cell Lung Cancer (EMERGING-CTONG 1103): A Randomized Phase II Study. J Clin Oncol (2019) 37:2235–45. 10.1200/jco.19.00075 31194613

[12] Xiong L Lou Y Bai H Li R Xia J Fang W. Efficacy of Erlotinib as Neoadjuvant Regimen in EGFR-mutant Locally Advanced non-Small Cell Lung Cancer Patients. J Int Med Res (2019) 48(4):300060519887275. 10.1177/0300060519887275 31885349 PMC7607055

[13] Tierney JF Stewart LA Ghersi D Burdett S Sydes MR. Practical Methods for Incorporating Summary Time-to-Event Data Into Meta-Analysis. Trials (2007) 8:16. 10.1186/1745-6215-8-16 17555582 PMC1920534

[14] Shi X Dong X Young S Chen AM Liu X Zheng Z. The Impact of Angiogenesis Inhibitors on Survival of Patients With Small Cell Lung Cancer. Cancer Med (2019) 8:5930–8. 10.1002/cam4.2462 PMC6792507 31433125

[15] Pisters KM Vallières E Crowley JJ Franklin WA Bunn PA Jr Ginsberg RJ. Surgery With or Without Preoperative Paclitaxel and Carboplatin in Early-Stage non-Small-Cell Lung Cancer: Southwest Oncology Group Trial S9900, an Intergroup, Randomized, Phase III Trial. J Clin Oncol (2010) 28:1843–9. 10.1200/jco.2009.26.1685 PMC2860367 20231678

[16] Lewis J Gillaspie EA Osmundson EC Horn L. Before or After: Evolving Neoadjuvant Approaches to Locally Advanced non-Small Cell Lung Cancer. Front Oncol (2018) 8:5. 10.3389/fonc.2018.00005 29410947 PMC5787144

[17] Zhou C Wu YL Chen G Feng J Liu XQ Wang C. Erlotinib Versus Chemotherapy as First-Line Treatment for Patients With Advanced EGFR Mutation-Positive non-Small-Cell Lung Cancer (OPTIMAL, CTONG-0802): A Multicentre, Open-Label, Randomised, Phase 3 Study. Lancet Oncol (2011) 12:735–42. 10.1016/s1470-2045(11)70184-x 21783417

