# Supplementary material for: Current Evidence of the Efficacy and Safety of Neoadjuvant EGFR-TKIs for Patients With Non-small Cell Lung Cancer
Source: Front Oncol. 2021 May 24;11:608608. doi: 10.3389/fonc.2021.608608 (PMC8181135; doi:10.3389/fonc.2021.608608)
Supplement: Supplementary file 1 [file DataSheet_1.pdf]

Current evidence of the efficacy and safety of neoadjuvant EGFR-TKIs for patients with non-small cell lung cancer (NSCLC)

Xiaoshun Shi, Xiaoying Dong, Jianxue Zhai, Xiguang Liu, Di Lu, Zhen Ni, Hua Wu, Kaican Cai

Department of Thoracic Surgery, Nanfang Hospital, Southern Medical University, Guangzhou 510515, China

Supplementary files

Table of Contents

Items	Description	Page
1	Literature search criteria	3
2	Figure S1 Proportional meta-analysis of pathological response rate	3
3	Figure S2 Proportional meta-analysis of the ORR in overall population including wild type EGFR status	4
4	Figure S3 Proportional meta-analysis of the rate of SD in population with EGFR-TKI-sensitive mutations	4
5	Figure S4 Meta regression analysis of the rate of SD in population with EGFR-TKI-sensitive mutations based on including early stage NSCLC or not.	4
6	Figure S5 Proportional meta-analysis of the rate of rash in population with wild type EGFR population	5
7	Figure S6 Meta regression analysis of the rate of rash in overall population based on patients with EGFR-TKIs sensitive mutation or not.	5
8	Figure S7 Proportional meta-analysis of the diarrhea of rash in population with wild type EGFR population	6
9	Figure S8 Meta regression analysis of the rate of diarrhea in overall population based on patients with EGFR-TKIs sensitive mutation or not.	6
10	Figure S9 Meta regression analysis of the rate of rash in overall population based on including early stage NSCLC or not.	7
11	Figure S10 Meta regression analysis of the rate of diarrhea in overall population based on including early stage NSCLC or not.	7
12	Figure S11 Funnel plot of surgical rate in population with EGFR-TKI-sensitive mutations(a), the ORR in population with EGFR-TKI-sensitive mutations(b), the ORR in overall population including wild type EGFR status(c), rate of pathological response in population with EGFR-TKI-sensitive mutations(d), rate of stable disease in population with EGFR-TKI-sensitive mutations(e), rate of grade 1-2 AEs in population with EGFR-TKI-sensitive mutations(f), rate of grade 3-4 AEs in population with EGFR-TKI-sensitive mutations(g), rate of grade 1-2 rash in population with EGFR-TKI-sensitive mutations(h), rate of grade 1-2 rash in overall population including wild type EGFR status(i), rate of grade 1-2 diarrhea in overall population including wild type EGFR status(j), and rate of grade 1-2 diarrhea in population with EGFR-TKI-sensitive mutations(k).	8-9

13	Table S1 Meta-Analysis of Observational Studies in Epidemiology (MOOSE) standards	10-11
14	Table S2 Quality assessment of included studies by using the Newcastle Ottawa Scale.	12
15	Table S3 The normality test of rates in proportional meta-analysis.	13

1.Literature search criteria

((((((((((((non-small-cell lung cancer[title] OR non-small cell lung cancer[title]) OR non small-cell lung cancer[title]) OR non small cell lung cancer[title]) OR non-small-cell lung carcinoma[title]) OR non-small cell lung carcinoma[title]) OR non small-cell lung carcinoma[title]) OR non small cell lung carcinoma[title]) OR nsclc[title]) AND (epidermal growth factor receptor[title/abstract] OR EGFR[title/abstract])) AND ((((((((((((((treatment[title/abstract] OR therapy[title/abstract]) OR tyrosine kinase inhibitor[title/abstract]) OR TKI[title/abstract]) OR osimertinib[title/abstract]) OR dacomitinib[title/abstract]) OR afatinib[title/abstract]) OR erlotinib[title/abstract]) OR gefitinib[title/abstract]) OR icotinib[title/abstract]) OR preoperative[title/abstract]) OR neoadjuvant[title/abstract]) OR surgery[title/abstract])) AND (English[Language])) AND ("0001/01/01"[Date - Publication] : "2020/05/11"[Date - Publication])

Supplementary figures

Figure S1 Proportional meta-analysis of pathological response rate

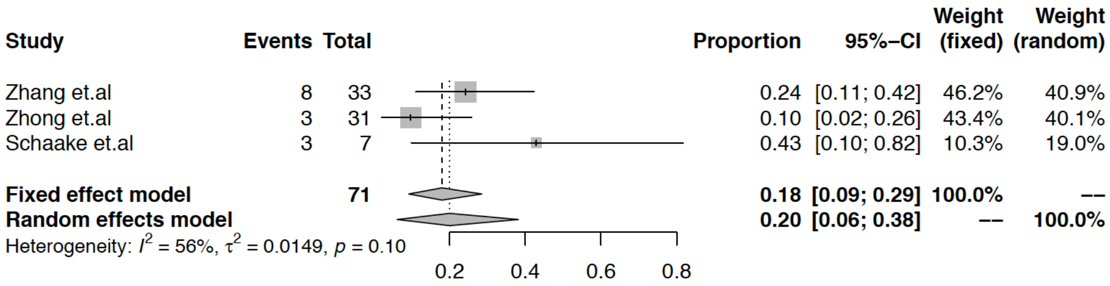

Figure S2 Proportional meta-analysis of the ORR in overall population including wild type EGFR status

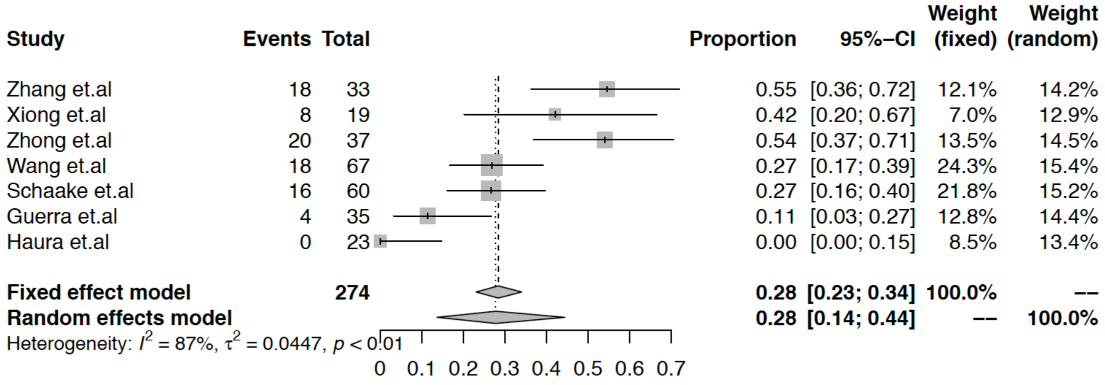

Figure S3 Proportional meta-analysis of the rate of SD in population with EGFR-TKI-sensitive mutations

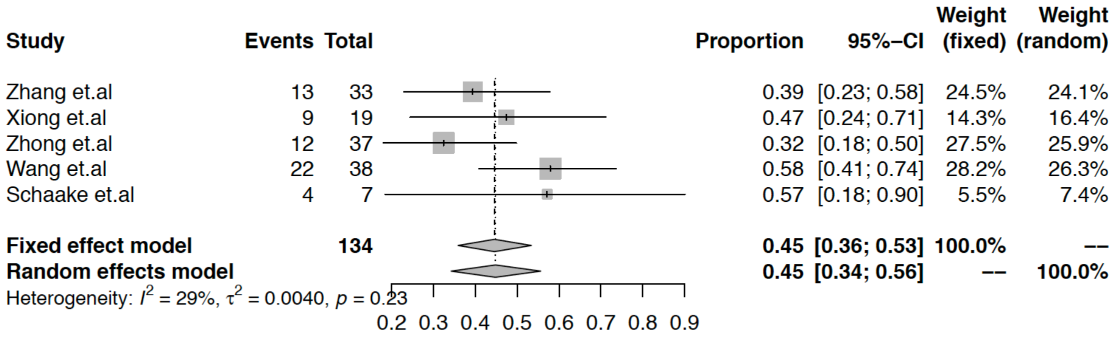

Figure S4 Meta regression analysis of the rate of SD in population with EGFR-TKI-sensitive mutations based on researches including early stage NSCLC or not.

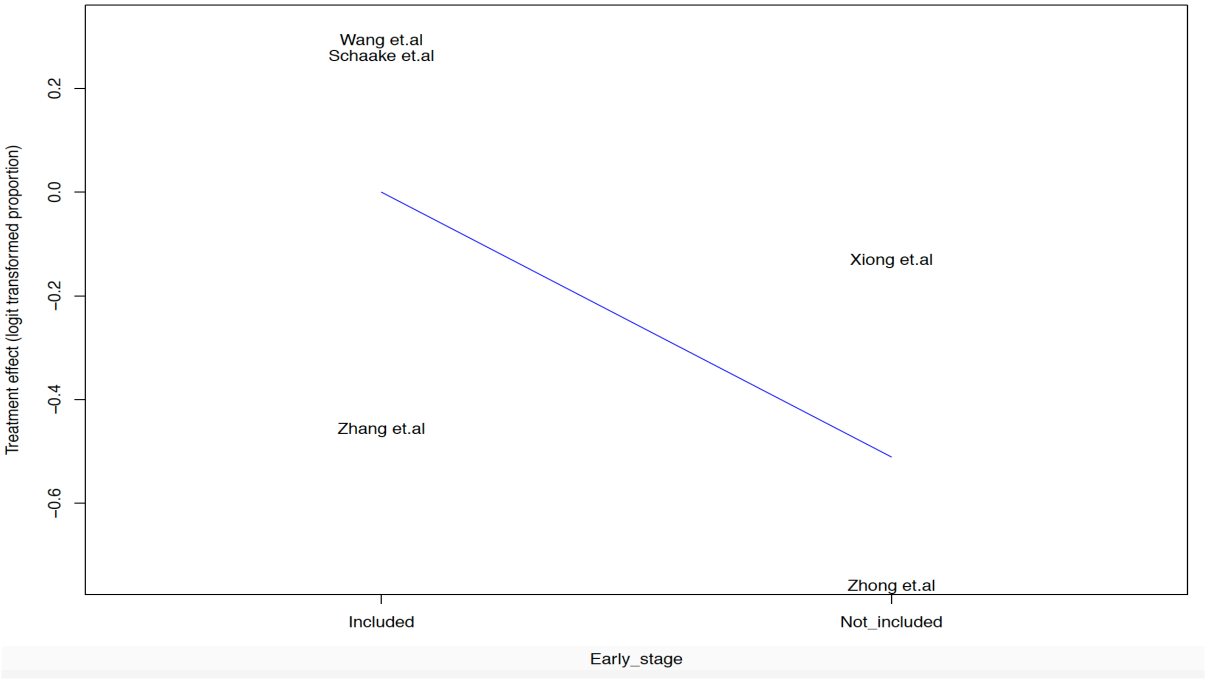

Figure S5 Proportional meta-analysis of the rate of rash in population with wild type EGFR population

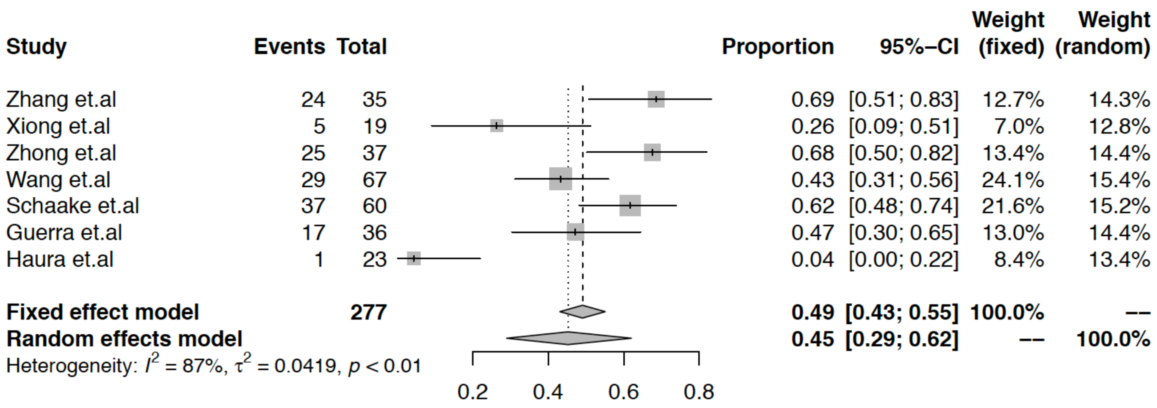

Figure S6 Meta regression analysis of the rate of rash in overall population based on patients with EGFR-TKIs sensitive mutation or not.

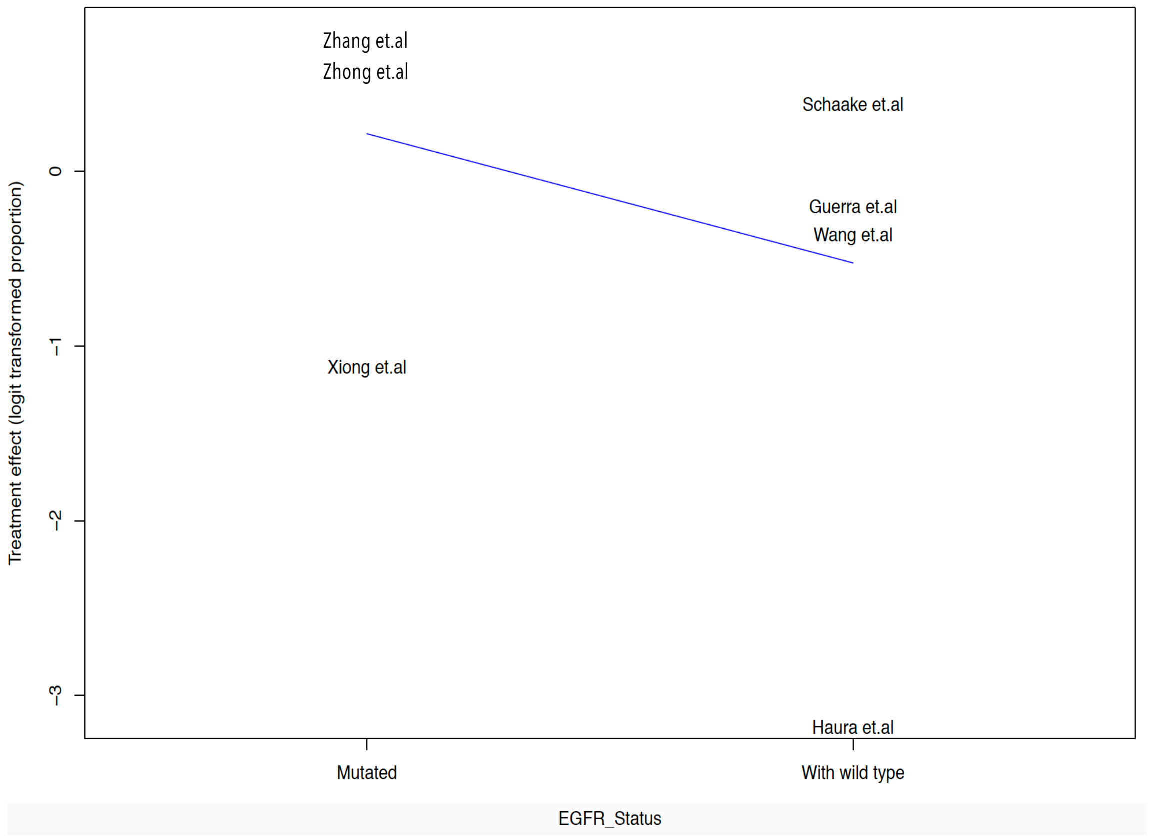

Figure S7 Proportional meta-analysis of the diarrhea of rash in population with wild type EGFR population

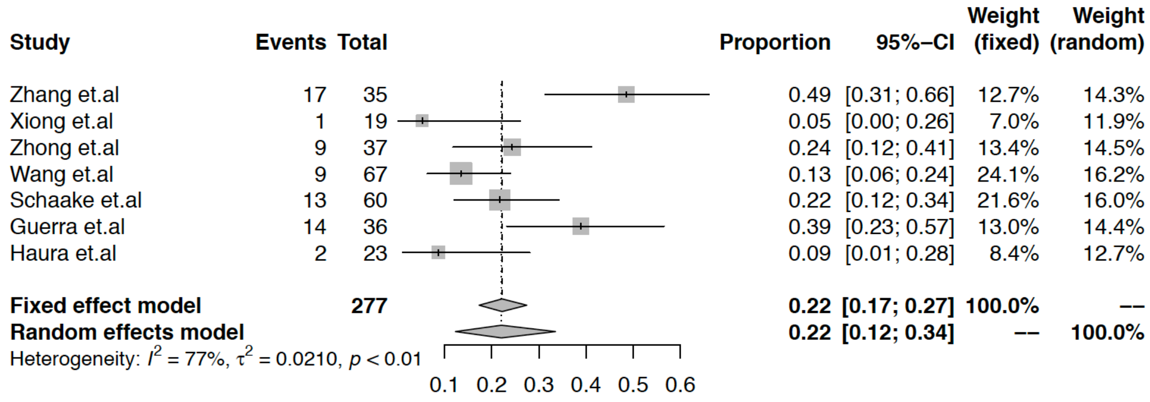

Figure S8 Meta regression analysis of the rate of diarrhea in overall population based on patients with EGFR-TKIs sensitive mutation or not.

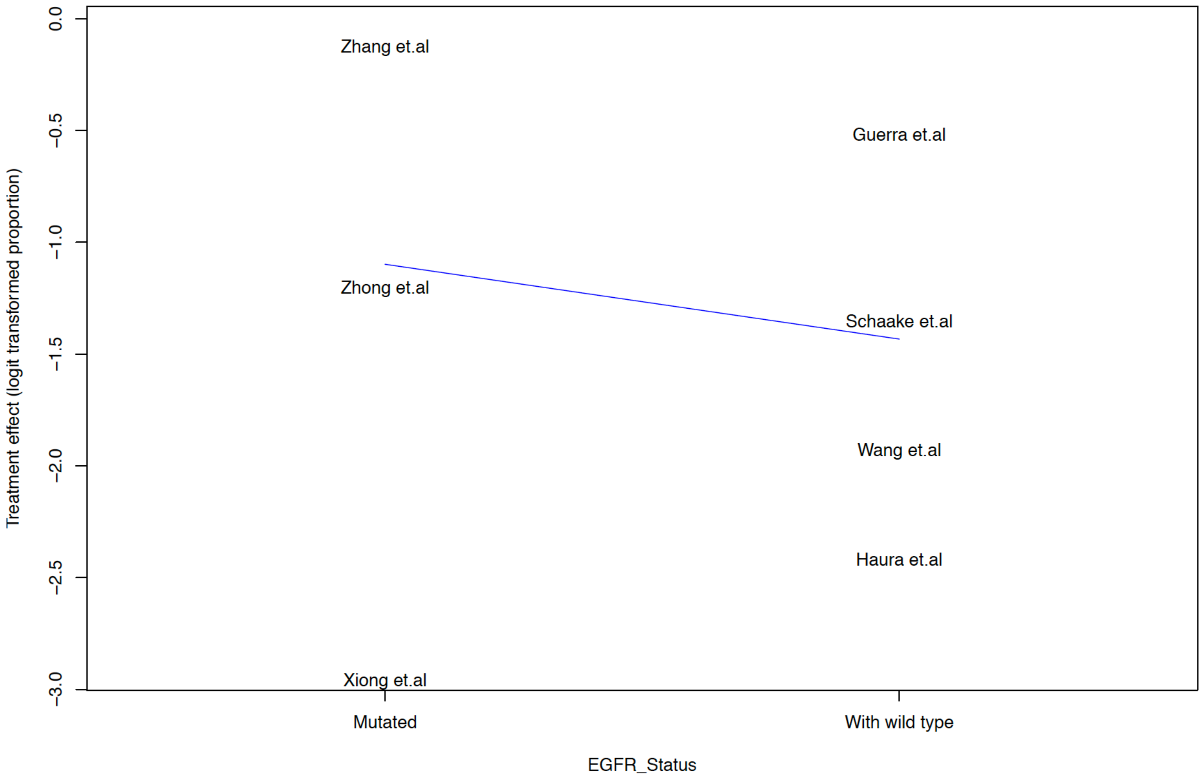

Figure S9 Meta regression analysis of the rate of rash in overall population based on including early stage NSCLC or not.

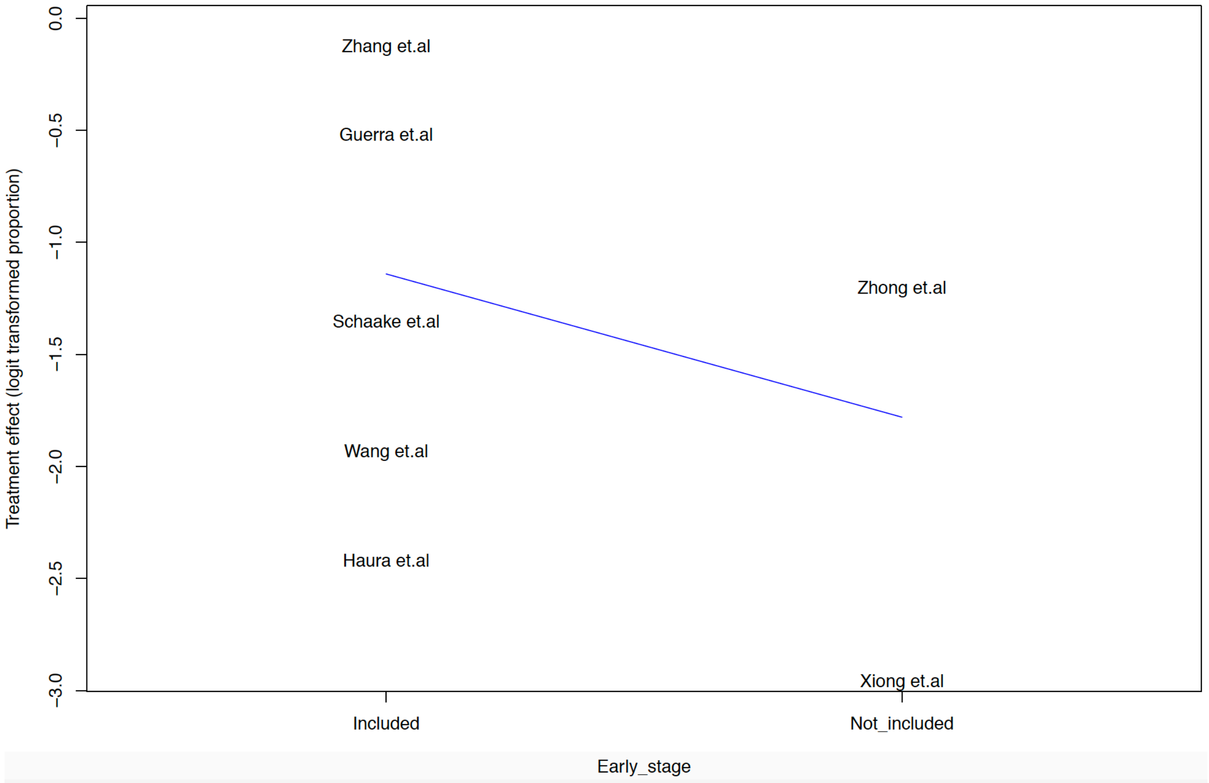

Figure S10 Meta regression analysis of the rate of diarrhea in overall population based on including early stage NSCLC or not.

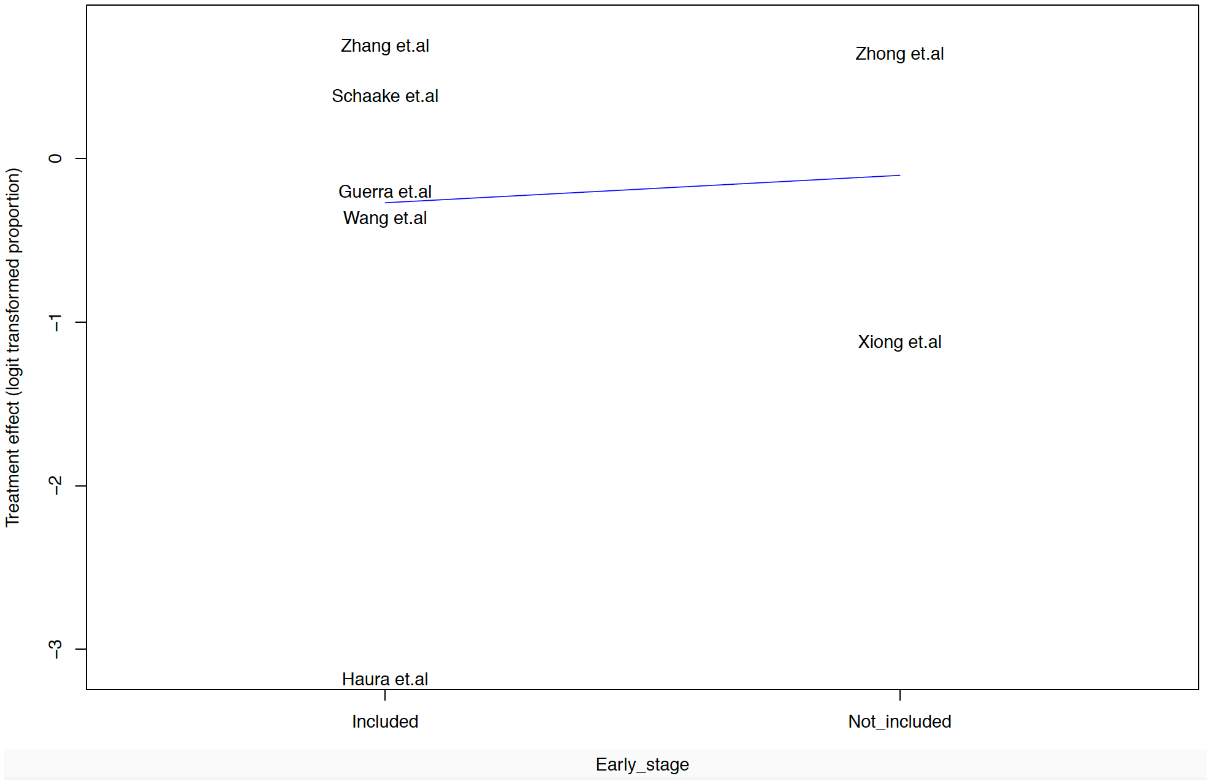

Figure S11 Analysis of publication bias. Funnel plot of surgical rate in population with EGFR-TKI-sensitive mutations(a, Egger's test: t = -1.2434, df = 5, p-value = 0.2689), the ORR in population with EGFR-TKI-sensitive mutations(b, Egger's test: t = -1.4595, df = 3, p-value = 0.2405), the ORR in overall population including wild type EGFR status(c, Egger's test: t = -0.17277, df = 5, p-value = 0.8696), rate of pathological response in population with EGFR-TKI-sensitive mutations(d, Egger's test: t = 0.91115, df = 1, p-value = 0.5296), rate of stable disease in population with EGFR-TKI-sensitive mutations(e, Egger's test: t = 0.47984, df = 3, p-value = 0.6642), rate of grade 1-2 AEs in population with EGFR-TKI-sensitive mutations(f, Egger's test: t = -2.5808, df = 1, p-value = 0.2353), rate of grade 3-4 AEs in population with EGFR-TKI-sensitive mutations(g, Egger's test: t = 1.0607, df = 3, p-value = 0.3667), rate of grade 1-2 rash in population with EGFR-TKI-sensitive mutations(h, Egger's test: t = -9.2561, df = 1, p-value = 0.06851), rate of grade 1-2 rash in overall population including wild type EGFR status(i, Egger's test: t = -1.2434, df = 5, p-value = 0.2689), rate of grade 1-2 diarrhea in overall population including wild type EGFR status(j, Egger's test: t = -0.074812, df = 5, p-value = 0.9433), and rate of grade 1-2 diarrhea in population with EGFR-TKI-sensitive mutations(k, Egger's test: t = -1.1618, df = 1, p-value = 0.4524).

A

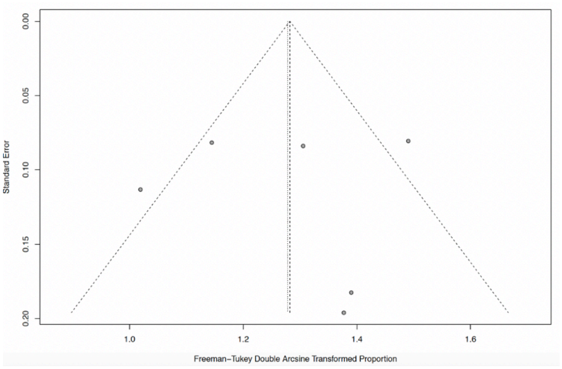

B

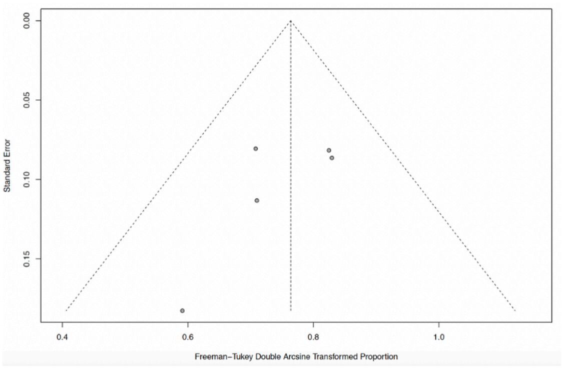

C

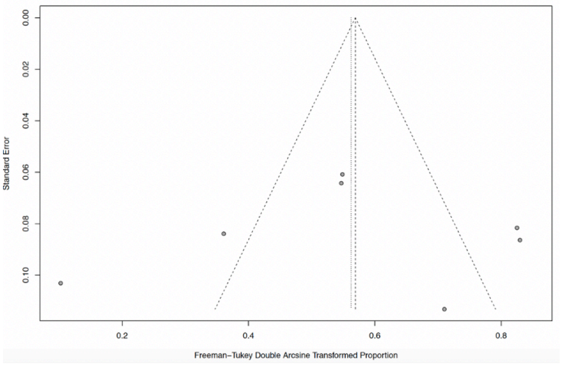

D

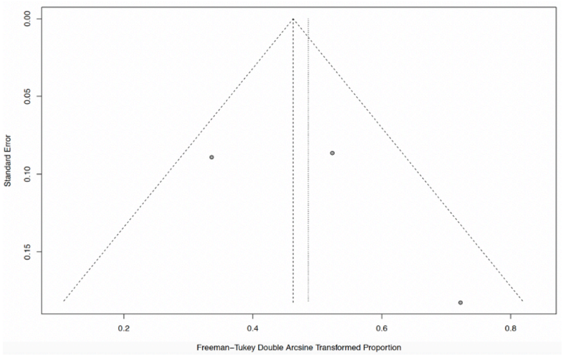

E

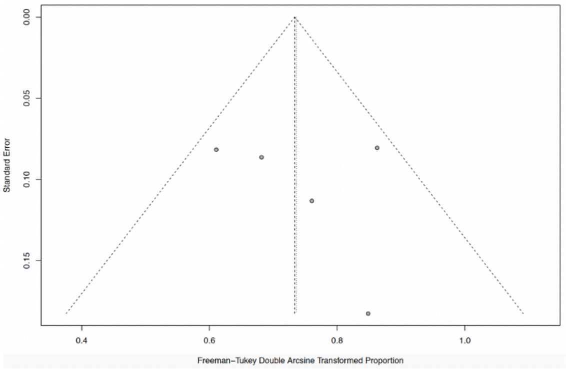
Standard Error
0.00
0.05
0.10
0.15
0.4
0.6
0.8
1.0
Freeman–Tukey Double Arcsine Transformed Proportion

F

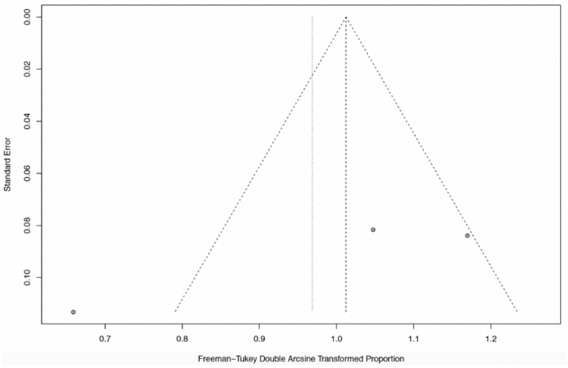
Standard Error
0.00
0.02
0.04
0.06
0.08
0.10
0.7
0.8
0.9
1.0
1.1
1.2
Freeman–Tukey Double Arcsine Transformed Proportion

G

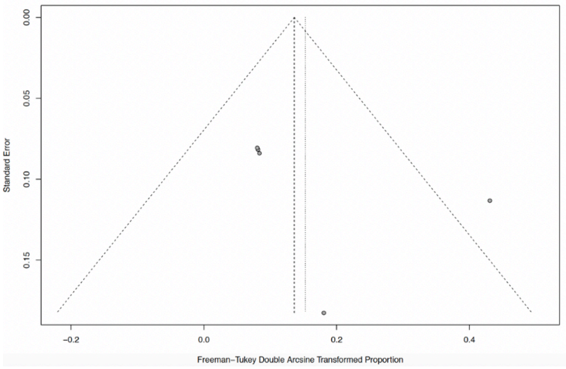
Standard Error
0.00
0.05
0.10
0.15
−0.2
0.0
0.2
0.4
Freeman–Tukey Double Arcsine Transformed Proportion

H

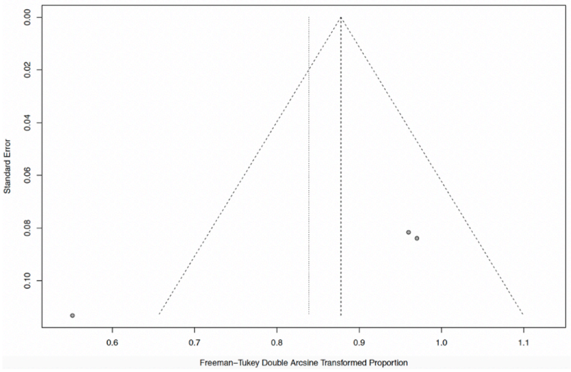
Standard Error
0.00
0.02
0.04
0.06
0.08
0.10
0.6
0.7
0.8
0.9
1.0
1.1
Freeman–Tukey Double Arcsine Transformed Proportion

I

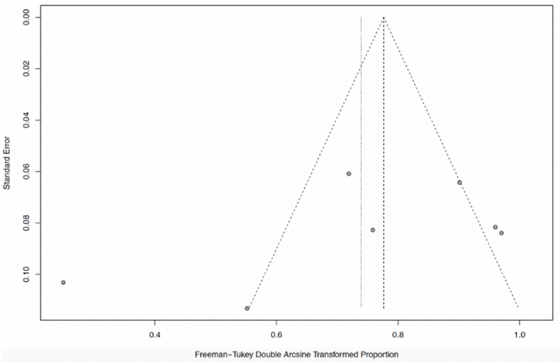
Standard Error
0.00
0.02
0.04
0.06
0.08
0.10
0.4
0.6
0.8
1.0
Freeman–Tukey Double Arcsine Transformed Proportion

J

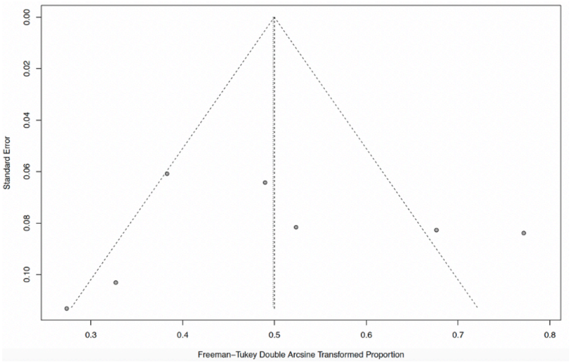
Standard Error
0.00
0.02
0.04
0.06
0.08
0.10
0.3
0.4
0.5
0.6
0.7
0.8
Freeman–Tukey Double Arcsine Transformed Proportion

K

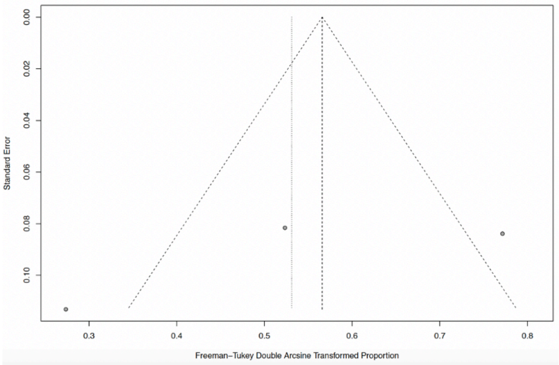
Standard Error
0.00
0.02
0.04
0.06
0.08
0.10
0.3
0.4
0.5
0.6
0.7
0.8
Freeman–Tukey Double Arcsine Transformed Proportion

Table S1. MOOSE (Meta-Analysis in Observational Studies in Epidemiology) Checklist

REPORTING CRITERIA	Reported	Page No.
**Reporting of Background**		
Problem definition	Yes	2,3
Hypothesis statement	Yes	3,4
Description of study outcomes(s)	Yes	2,3
Type of exposure or intervention used	Yes	2,3,4
Type of study design used	Yes	2,3,4
Study population	Yes	2,3,4
**Reporting of Search Strategy**		
Qualifications of searchers (librarians & investigators)	Yes	4,5
Search strategy including time period included in synthesis and keywords	Yes	4,5
Effort to include all available studies including contact with authors	Yes	4,5
Database and registries searched	Yes	4,5
Search software used, name and version, including special features used	Yes	5,6
Use of hand searching (e.g. reference list of obtained articles)	Yes	NA
List of citations located and those excluded, including justification	Yes	5
Method for addressing articles published in languages other than English	Yes	5
Method of handling abstracts and unpublished studies	Yes	5,6
Description of any contact with authors	Yes	5,6
**Reporting of Methods**		
Description of relevance or appropriateness of studies assembled for assessing the hypothesis to be tested	Yes	4,5,6
Rationale for the selection and coding of data (e.g. sound clinical principles or convenience)	Yes	4,5,6
Documentation of how data were classified and coded (e.g. multiple raters, blinding, and interrater reliability)	Yes	4,5,6
Assessment of confounding (e.g. comparability of cases and controls in studies where appropriate)	Yes	4,5,6
**Reporting Criteria**		
Assessment of study quality, including blinding of quality assessors; stratification or regression of possible predictors of study results	Yes	4,5,6
Assessment of heterogeneity	Yes	4,5,6
Description of statistical methods (e.g. complete description of fixed or random effects models, justification of whether chosen models account for predictors of study results, dose response models or cumulative meta-analysis) in sufficient detail to be replicated	Yes	4,5,6
Provision of appropriate tables or graphs	Yes	11,12
**Reporting of Results**		
Table giving descriptive information for each study included	Yes	6
Results of sensitivity testing (e.g. subgroup analysis)	Yes	6,7,8
Indication of statistical uncertainty of findings	Yes	8
**Reporting of Discussion**		
Quantitative assessment of bias	Yes	8,9

Justification of exclusion	Yes	8,9
Assessment of quality of included studies	Yes	8,9
**Reporting of Conclusions**		
Consideration of alternative explanations for observed results	Yes	8,9
Generalisation of conclusions	Yes	10
Guidelines for future research	Yes	10
Disclosure of funding source	Yes	11

Table S2 Quality assessment of included studies by using the Newcastle Ottawa Scale.

	**Selection**			**Comparability (**)**	**Outcome**			**Total (7*)**	**Quality Score**
	Representativeness of exposed cohort (*)	Selection of non-exposed cohort (⋆)	Ascertainment of exposure (⋆)		Assessment of outcome (*)	Adequacy of length of follow up (*)	Adequacy of completeness of follow up (*)		
**Zhang et al. 2020** *The Journal of Thoracic and Cardiovascular Surgery*	*	*	*	-	*	*	*	6	Good
**Xiong et al. 2018** *The Oncologist*	*	*	*	*	*	*	*	7	Good
**Zhong et al. 2019** *Journal of Clinical Oncology*	*	*	*	*	*	*	*	7	Good
**Wang et al. 2016** *OncoTargets and Therapy*	*	*	*	*	-	-	-	4	Poor
**Schaake et al. 2012** *Journal of Clinical Oncology*	*	*	*	*	-	-	-	4	Poor
**Lara-Guerra et al. 2009** *Journal of Clinical Oncology*	-	*	*	*	*	*	*	6	Good
**Haura et al. 2010** *Journal of Thoracic Oncology*	*	-	*	-	*	-	-	4	Poor

Table S3 The normality test of rates in proportional meta-analysis.

Rates	Raw	Log transformation	Logit transformation	Arcsin transformation	Double arcsin transformation
Surgical rate	W = 0.80471, p-value = 0.06476	W = 0.79664, p-value = 0.05481	W = NaN, p-value = NA	W = 0.99089, p-value = 0.9912	W = 0.81867, p-value = 0.08594
ORR in patients with EGFR-TKIs sensitive mutation	W = 0.88796, p-value = 0.347	W = 0.86749, p-value = 0.2564	W = 0.88411, p-value = 0.3284	W = 0.88412, p-value = 0.3284	W = 0.91982, p-value = 0.5288
Pathological response	W = 0.99506, p-value = 0.8656	W = 0.98204, p-value = 0.7433	W = 0.99485, p-value = 0.8629	W = 0.99502, p-value = 0.865	W = 0.98953, p-value = 0.8042
Grade 1-2 Adverse effects	W = 0.89629, p-value = 0.3738	W = 0.85789, p-value = 0.2618	W = 0.9403, p-value = 0.5286	W = 0.91045, p-value = 0.4196	W = 0.92154, p-value = 0.4578
Grade 3-4 Adverse effects	W = 0.55218, p-value = 0.000131	W = NaN, p-value = NA	W = NaN, p-value = NA	W = 0.55218, p-value = 0.000131	W = 0.68361, p-value = 0.006403
Stable disease	W = 0.91475, p-value = 0.4966	W = 0.91379, p-value = 0.4907	W = 0.91647, p-value = 0.5074	W = 0.98929, p-value = 0.9771	W = 0.98019, p-value = 0.9356
ORR with wild type EGFR population	W = 0.93057, p-value = 0.5558	W = NaN, p-value = NA	W = NaN, p-value = NA	W = 0.89884, p-value = 0.324	W = 0.90387, p-value = 0.355
Diarrhea in patients with EGFR-TKIs sensitive mutation	W = 0.99524, p-value = 0.8682	W = 0.95462, p-value = 0.59	W = 0.98129, p-value = 0.738	W = 0.99869, p-value = 0.9308	W = 0.99988, p-value = 0.9788
Diarrhea in overall patients	W = 0.93328, p-value = 0.5792	W = 0.96472, p-value = 0.8581	W = 0.97603, p-value = 0.9382	W = 0.96447, p-value = 0.8561	W = 0.95006, p-value = 0.7302
Rash in patients with EGFR-TKIs sensitive mutation	W = 0.76781, p-value = 0.03976	W = 0.76155, p-value = 0.02567	W = 0.76913, p-value = 0.04275	W = 0.7661, p-value = 0.03591	W = 0.77344, p-value = 0.05255
Rash in overall patients	W = 0.90668, p-value = 0.3734	W = 0.7312, p-value = 0.00804	W = 0.83368, p-value = 0.0867	W = 0.87635, p-value = 0.2107	W = 0.89136, p-value = 0.2818

- Red cells indicated the rates do not follow normal distribution. We used the Freeman-Tukey double arcsine transformation for it has the greatest number of normal distributed rates (10 out of 11) across the methods (green cells).
